# *Pneumocystis jirovecii* Pneumonia Diagnosis with Oropharyngeal Wash PCR in Immunocompromised Patients—A Systematic Review

**DOI:** 10.3390/jcm14186572

**Published:** 2025-09-18

**Authors:** Vasco Salgado Costa, José Pedro Cidade, Inês Medeiros, Pedro Fidalgo, Hugo Moreira, Teresa Miranda, Pedro Póvoa

**Affiliations:** 1 Department of Critical Care Medicine, Hospital de São Francisco Xavier, Unidade Local de Saúde Lisboa Ocidental (ULSLO), Estrada Forte do Alto Duque, 1449-005 Lisbon, Portugal; zencidade@gmail.com (J.P.C.); imedeiros@ulslo.min-saude.pt (I.M.); pedrofidalgo.com@gmail.com (P.F.); hugomnm@gmail.com (H.M.); teresablmiranda@gmail.com (T.M.); pedrorpovoa@gmail.com (P.P.); 2 NOVA Medical School, New University of Lisbon, 1169-056 Lisbon, Portugal; 3 Centre for Clinical Epidemiology and Research Unit of Clinical Epidemiology, OUH Odense University Hospital, DK-5230 Odense, Denmark; 4 D’Or Institute for Research and Education (IDOR), Rio de Janeiro 22281-100, RJ, Brazil

**Keywords:** *Pneumocystis jirovecii* pneumonia, oropharyngeal wash, polymerase chain reaction, non-invasive diagnosis, immunocompromised

## Abstract

**Background/Objectives:**  *Pneumocystis jirovecii* pneumonia (PJP) remains a major cause of morbidity and mortality in immunocompromised patients. Bronchoalveolar lavage (BAL) is the diagnostic gold standard but is invasive and often impractical in critically ill patients. Oropharyngeal wash (OW) polymerase chain reaction (PCR) offers a rapid, non-invasive alternative. We performed a systematic review focusing on this respiratory sample’s diagnostic accuracy and clinical utility. **Methods:** We searched PubMed, Scopus, Web of Science, Cochrane Library, and clinical trial registries including ClinicalTrials.gov and MedRxiv for studies of PCR-based *P. jirovecii* detection in OW samples from immunocompromised adults, using BAL or induced sputum as reference standards. The Preferred Reporting Items for Systematic Reviews and Meta-Analyses (PRISMA) methodology was followed. Quality was assessed with Quality Assessment of Diagnostic Accuracy Studies-2 (QUADAS-2), and pooled sensitivity/specificity were estimated using a bivariate random-effects model. **Results:** Twelve studies (*n* = 633; 346 confirmed PJP cases) met the inclusion criteria. Most cohorts were human immunodeficiency virus (HIV)-positive. Pooled sensitivity was 68.3% (95% CI: 59.2–75.9) and specificity 91.8% (95% CI: 85.9–95.3); the area under the summary receiver operating characteristic curve (AUC) was 0.887. Diagnostic yield improved with pre-sample cough induction, 60-s gargling, early sampling before extended therapy, and higher fungal loads. Risk of bias was low, and no significant publication bias was detected. **Conclusions:** OW-based PCR delivers high specificity and moderate sensitivity for PJP diagnosis, offering a safe, scalable, and patient-friendly alternative when invasive testing is unfeasible. Optimizing collection protocols and expanding evaluation to non-HIV immunosuppressed populations could enhance its role as an early screening tool, enabling faster treatment decisions and reducing unnecessary antimicrobial exposure.

## 1. Introduction

*Pneumocystis jirovecii,* formerly known as *P. carinii*, is an opportunistic fungal pathogen responsible for Pneumocystis pneumonia (PJP), a life-threatening infection that primarily affects immunocompromised individuals [1,2]. Once misclassified as a protozoan, *P. jirovecii* is now recognized as an ascomycetous fungus with unique biological properties. Its clinical significance became particularly evident during the human immunodeficiency virus/acquired immunodeficiency syndrome (HIV/AIDS) epidemic, when it emerged as a major cause of respiratory failure and mortality [2].

PJP typically occurs in patients with profound cellular immunosuppression, including those with HIV, hematologic malignancies, solid organ transplants, or receiving corticosteroids or other immunosuppressive therapies [2,3]. In recent decades, the epidemiology of PJP has gradually shifted from primarily affecting the HIV population to now targeting other immunosuppressed patients. These changes are mostly due to advancements in antiretroviral therapies for HIV and adequate prophylaxis of PJP and the parallel and broader use of immunomodulators in non-HIV immunosuppressed patients [1,3]. Many of the clinical practices for this population are derived from HIV patients; nonetheless, many knowledge gaps remain to be answered, namely prophylaxis duration and indications, risk stratification and treatment [1,4].

This disease often presents as a subacute respiratory illness with fever, non-productive cough, and progressive dyspnoea [3,4]. In high-risk populations, especially HIV-negative individuals, PJP can rapidly progress to acute hypoxemic respiratory failure, frequently necessitating ICU admission and invasive mechanical ventilation. Mortality remains substantial, ranging from 10–20% in HIV-positive patients to 30–50% in HIV-negative individuals, with delayed diagnosis, atypical presentations, and a heightened inflammatory response contributing to poorer outcomes in the latter group [4,5,6].

Despite its clinical impact, diagnosing PJP in critically ill patients remains challenging. The gold standard involves detection of *P. jirovecii* in respiratory specimens, ideally bronchoalveolar lavage (BAL), through microscopic staining or molecular methods such as quantitative PCR (qPCR) [7,8,9]. However, BAL is frequently infeasible in unstable patients due to its invasiveness and associated risks, including respiratory deterioration and the need for intubation. Less invasive alternatives, such as induced sputum and oropharyngeal wash (OW), have been explored, but their diagnostic performance in ICU settings remains incompletely defined. Serum biomarkers such as (1,3)-β-D-glucan (BDG) and lactate dehydrogenase (LDH) are commonly used as adjunctive tools but offer limited specificity [3,10]. Although BDG shows moderate sensitivity, particularly in HIV-positive patients, it lacks sufficient diagnostic accuracy in non-HIV immunosuppressed populations to be used as a standalone test [10]. As a result, microbiological confirmation via BAL remains the diagnostic mainstay [1,3,4], despite its practical limitations in critical care.

Advances in real-time PCR (RT-PCR) and qPCR have markedly improved the sensitivity of *P. jirovecii* detection, even in low-burden infections. When applied to oropharyngeal wash samples, these molecular techniques may offer a rapid, non-invasive, and scalable diagnostic alternative especially valuable in critically ill patients unable to tolerate invasive procedures. However, variation in sampling methods, PCR targets, and interpretation thresholds across studies has hindered widespread clinical adoption. The diagnostic performance of oropharyngeal wash PCR in non-HIV immunocompromised patients, a group with delayed diagnoses and higher mortality, remains unclear.

This systematic review aims to synthesize current evidence on the use of oropharyngeal wash-based PCR for the diagnosis of PJP in immunocompromised individuals, specifically in terms of (1) diagnostic accuracy; (2) feasibility and utility in critically ill patients; and (3) potential to substantiate timely and safe interventions in the ICU. In an era of precision and rapid diagnostics, addressing this evidence gap is crucial to improving outcomes in vulnerable patient populations.

## 2. Materials and Methods

### 2.1. Search Strategy and Data Sources

We conducted a systematic review based on a comprehensive literature search performed between May and June 2025, aimed at identifying studies evaluating the detection of *Pneumocystis jirovecii* by PCR in oropharyngeal wash samples of immunocompromised adults. Studies published from 1990 to June 2025, and written in English, were considered eligible. The search was conducted across the following databases: PubMed, Scopus, Web of Science, Cochrane Library, and clinical trial registries, including ClinicalTrials.gov and MedRxiv. A predefined search strategy was developed as part of a review protocol, following the Preferred Reporting Items for Systematic Reviews and Meta-Analyses (PRISMA) guidelines. The protocol was prospectively registered in the PROSPERO database (Registration ID: CRD420251059384).

### 2.2. Eligibility Criteria

Studies were eligible for inclusion if they met all of the following criteria: (1) Included adult patients (≥18 years) who were immunocompromised due to an underlying disease or immunosuppressive therapy; (2) Employed PCR-based detection of *Pneumocystis jirovecii* in oropharyngeal wash samples, with or without other oropharyngeal samples; (3) Diagnosed PJP according to EORTC, CDC/NIH/IDSA, or EACS (European AIDS Clinical Society) guidelines, as clinically indicated.

The following exclusion criteria were applied: (1) Studies involving paediatric or immunocompetent populations; (2) PCR detection from lower respiratory tract samples only (e.g., spontaneous or induced sputum, mini-bronchoalveolar lavage [mini-BAL], or bronchoalveolar lavage [BAL]); (3) Non-original research, including reviews, systematic reviews, meta-analyses, editorials, opinion pieces, comments, conference abstracts, case reports with fewer than 10 patients (defined for the statistical power required for the diagnostic accuracy study intended in this review, limiting the diagnostic accuracy discrepancies in smaller-numbered studies), or studies with insufficient data; (4) Studies published before 1990, after June 2025, or in languages other than English.

### 2.3. Study Selection and Data Extraction

Screening and data extraction were conducted independently and in random order by three investigators using the Rayyan^®^ platform, including duplicate screening. All discrepancies were mitigated by an external expert on this topic, thus deciding on the inclusion or not of the study in the review. The following data were extracted: author(s), year of publication, study design, sample size, HIV status, antiretroviral therapy status and adherence, presence and type of other immunosuppressive therapies, clinical and radiological criteria for PJP diagnosis, PCR methodology (qualitative or quantitative with cycle threshold values), use of oropharyngeal wash and other oropharyngeal samples, oropharyngeal wash sample collection method as well as sample storage and transportation, safety measures and complications, beta-D-glucan serum and respiratory sample testing, and diagnostic accuracy parameters (true positives, false positives, true negatives, false negatives).

### 2.4. Quality Assessment

The methodological quality and risk of bias of included studies were assessed using the Quality Assessment of Diagnostic Accuracy Studies 2 (QUADAS-2) tool. Three reviewers independently evaluated each study across four domains: patient selection, index test, reference standard, and flow and timing. Disagreements were resolved by consensus. The outcomes of the quality assessment were used to guide the interpretation of results and to perform sensitivity analyses. Studies identified as having a high risk of bias in key domains were evaluated separately to assess their potential impact on pooled estimates. The customized QUADAS-2 Checklist for review and the risk of bias assessment table are both included in the Electronic Supplementary Materials, as Tables S7 and S8, respectively.

### 2.5. Data Synthesis

A qualitative synthesis was performed to summarize the key characteristics and findings of the included studies. For the quantitative synthesis, we conducted a meta-analysis of diagnostic accuracy using a bivariate random-effects model (Reitsma model) to estimate pooled sensitivity and specificity, accounting for between-study heterogeneity. Summary receiver operating characteristic (ROC) curves were generated to visualize diagnostic performance. Diagnostic accuracy data from each study (TP, FP, FN, TN) were used as input.

Heterogeneity was assessed using the I^2^ statistic, calculated separately for sensitivity and specificity based on the variance components of the bivariate model. Forest plots were visually inspected for heterogeneity across studies. Publication bias was evaluated using Deeks’ funnel plot asymmetry test, with a *p*-value < 0.05 considered indicative of potential bias. All statistical analyses were conducted using the ‘mada’ package in R (R Foundation for Statistical Computing, Version 4.4.0, Vienna, Austria). A two-sided *p*-value of <0.05 was considered statistically significant.

## 3. Results

**Figure 1:**
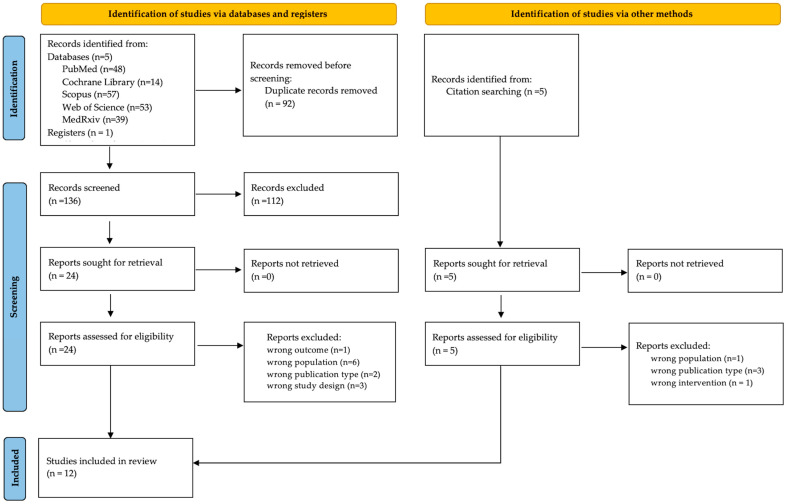
PRISMA Flowchart demonstrating the performed literature search for the systematic review. *n*—number.

The literature search yielded 228 records, of which 92 duplicates were removed, leaving 136 publications for title and abstract screening. Following the application of inclusion and exclusion criteria, 112 records were excluded, primarily due to irrelevant outcomes, inappropriate study populations, unsuitable publication types, or incompatible study designs. A total of 24 articles proceeded to full-text screening by all investigators (see PRISMA diagram, Figure 1). An additional five studies were identified through manual searching; however, all were excluded based on predefined criteria. Ultimately, 12 studies were included in the systematic review. No exclusions were made through automation tools. A concise version of the included studies’ information is displayed in Table 1. The full search strategy details, as well as a detailed summary of the included studies is provided in the Electronic Supplementary Materials Tables S1–S5 and Table S6, respectively.

### 3.1. General Characteristics of Included Studies

The 12 studies included in this review [11,12,13,14,15,16,17,18,19,20,21,22] were published between 1996 and 2016, the majority of which (83.3%, *n* = 10) employed a prospective observational design. Across all studies, a total of 633 patients were evaluated, of whom 346 were immunocompromised patients with confirmed *Pneumocystis jirovecii* pneumonia (PJP). Diagnostic confirmation of PJP varied between studies and included: Microscopic identification of *P. jirovecii* in bronchoalveolar lavage (BAL) [11,12,13,14,18,19,20,21] or induced sputum samples [15,17,18,20,21], and PCR-based detection in BAL [16] or induced sputum [20,22].

All included studies focused on immunocompromised adult populations, as defined by the inclusion criteria. Most patients were HIV-positive, with only two studies (16.7%, *n* = 2) [16,22] including individuals with non-HIV-related immunosuppression, such as solid tumours, hematologic malignancies, or autoimmune diseases. Only one study [13] reported CD4+ lymphocyte counts or HIV staging, based on CDC criteria. Two studies [19,21] mentioned whether patients were receiving antiretroviral therapy (ART), while just one [16] referenced non-ART immunosuppressive treatments, specifically systemic corticosteroids. None of the studies assessed or reported medication adherence or compliance.

### 3.2. Oropharyngeal Wash Collection and Detection of Pneumocystis jirovecii by PCR

Across the studies included in this systematic review, the diagnostic utility of oropharyngeal wash samples for the detection of *Pneumocystis jirovecii* was assessed using a variety of PCR-based methodologies. Special attention was given to pre-analytical factors potentially influencing test sensitivity.

#### 3.2.1. Reference Standards

Most of the included studies (*n* = 10) used bronchoalveolar lavage (BAL) as the reference standard to evaluate the diagnostic accuracy of OW samples [11,12,13,14,16,17,18,19,20,21]. This choice is consistent with current clinical practice, given BAL’s high diagnostic yield in PJP. Only two studies used induced sputum as the sole comparator [15,22].

#### 3.2.2. OW Sample Collection Protocols

All studies employed sterile saline for OW collection, with 10 mL being the most frequently employed target volume [11,12,13,14,15,16,17,18,19,20,21,22]. One study [19] allowed a range of 10–20 mL, adjusting to individual patient tolerance. Gargling duration varied considerably between studies, from a minimum of 5 s to a maximum of 2 min, although a 60-s gargle was the most commonly applied duration [14,15,17,18,20,21].

The study by Huang et al. specifically assessed the influence of both gargling duration and technique [17]. It demonstrated that inadequate gargling, regardless of duration, led to false-negative results, particularly in patients with low fungal burdens. Moreover, a gargling duration of 60 s yielded greater sensitivity compared to 40 s, highlighting the role of standardized technique in optimizing diagnostic yield.

**Figure 2:**
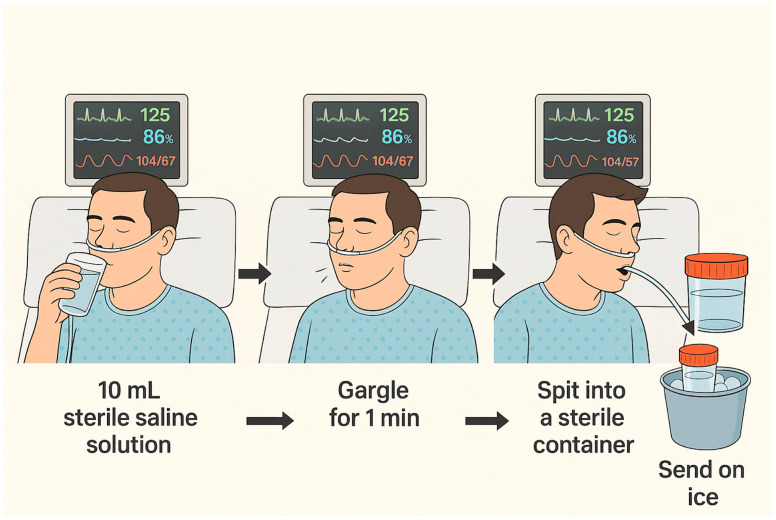
Suggested technique for performing the collection of an oropharyngeal wash sample. Min—minutes; mL—millilitres.

The technique depicted in Figure 2 illustrates the most consensual method for sample collection.

#### 3.2.3. Factors Influencing Diagnostic Sensitivity

Several pre-analytical factors were identified across the included studies as influencing the sensitivity of OW-based PCR testing. According to Huang et al., the following variables were associated with improved detection rates: Pre-sample cough induction, promoting mobilization of lower respiratory tract secretions; Prolonged and effective gargling, with 60 s being superior to shorter durations; Timing of collection, with samples obtained earlier in the disease course—prior to extended anti-PJP treatment—showing higher positivity rates [17].

These findings were further supported by studies utilizing quantitative PCR (qPCR), which showed higher detection rates in patients with greater fungal loads and shorter durations of prior treatment [18].

#### 3.2.4. Safety and Feasibility

No adverse events or complications related to OW collection were reported in any of the included studies, reinforcing the excellent safety profile of this procedure. In contrast to BAL, OW is non-invasive, simple, well-tolerated, and repeatable, representing a significant clinical advantage.

#### 3.2.5. Sample Handling, Storage, and Transport

Regarding transport, only one study [17] explicitly stated that OW samples were transported on dry ice. The remaining studies did not report transport conditions [11,12,13,14,15,16,18,19,20,21,22]. Sample storage conditions were inconsistently reported and varied depending on the molecular target. Among DNA-based studies [11,12,13,14,15,16,18,19,21], only four explicitly reported storage temperatures: −20 °C [13], −70 °C [8], 4 °C [19], −80 °C [21]. The remaining DNA-based studies did not mention storage details.

Three studies targeted RNA [17,20,22]. Among these, two specified storage at −70 °C [17,20], while one [22] did not report storage conditions. These findings suggest that when RNA is the molecular target, ultra-low temperature storage (−70 °C) is preferred to prevent degradation. Overall, the lack of standardized reporting on transport and storage practices may impact the reproducibility and reliability of PCR results, particularly for RNA-based assays.

### 3.3. Diagnostic Accuracy and Risk of Bias Assessment

We performed a quantitative synthesis of diagnostic performance across the twelve studies evaluating PCR-based detection of *Pneumocystis jirovecii* in oropharyngeal wash samples of immunocompromised adults.

**Figure 3:**
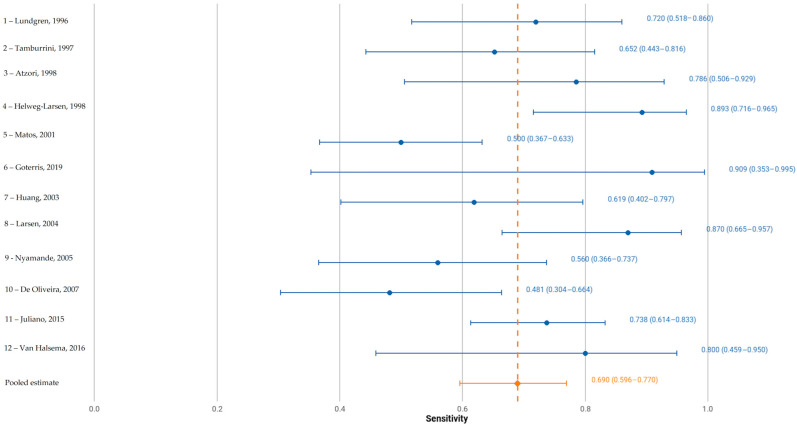
Forest plot of sensitivity of the included studies, where numbers 1 to 12 correspond to studies [11,12,13,14,15,16,17,18,19,20,21,22], respectively. Point estimates and 95% confidence intervals per study and pooled.

**Figure 4:**
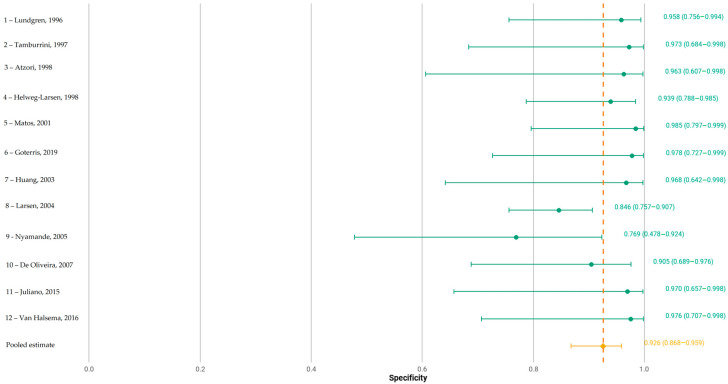
Forest plot of specificity of the included studies, where numbers 1 to 12 correspond to studies [11,12,13,14,15,16,17,18,19,20,21,22], respectively. Point estimates and 95% confidence intervals per study and pooled.

The pooled sensitivity was 0.683 (95% CI: 0.592–0.759) and pooled false-positive rate was 0.082 (95% CI: 0.047–0.141), corresponding to a pooled specificity of 0.918 (95% CI: 0.859–0.953), as depicted in Figure 3 and Figure 4, respectively.

**Figure 5:**
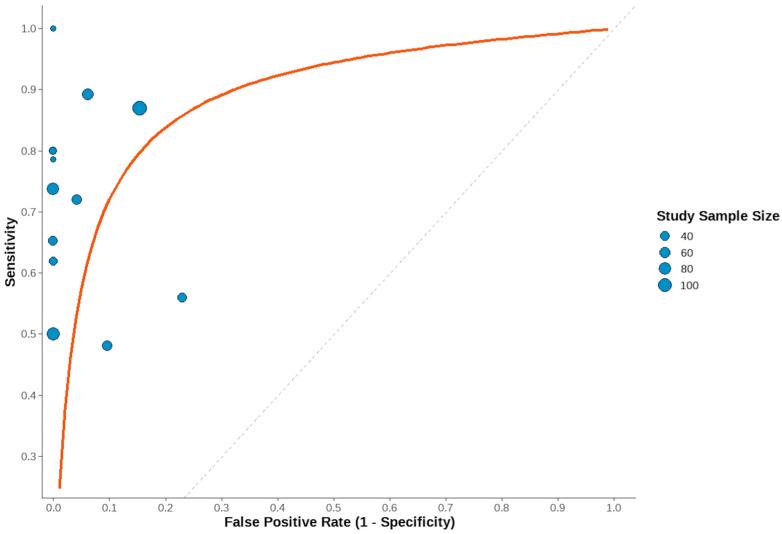
Receiver operating characteristic (ROC) curve (Rutter-Gatsonis Model) for the present systematic review of the twelve studies [11,12,13,14,15,16,17,18,19,20,21,22], with a high overall diagnostic accuracy.

The area under the summary receiver operating characteristic curve (AUC) was 0.887, indicating a high overall diagnostic accuracy. The normalized partial AUC, restricted to the observed false-positive rate range, was 0.698, reflecting moderate performance in the most clinically relevant specificity domain. A consistent sensitivity–specificity trade-off was evident across studies (Figure 5).

Between-study heterogeneity was moderate. The Zhou and Dendukuri I^2^ estimate was 13%, suggesting limited heterogeneity. However, unadjusted Holling’s I^2^ ranged from 32.7% to 44.8%, with adjusted values between 3% and 3.4%, reflecting some variability in effect estimates. Forest plot inspection confirmed variation in individual study sensitivity and specificity, although no single outlier was identified as a major source of heterogeneity.

Risk of bias, assessed using QUADAS-2 tool, was generally low across most domains. High risk was noted in patient selection in four studies, primarily due to case–control designs or poorly defined inclusion criteria. The index test was low risk in nearly all studies, with only one study rated high due to limited reporting on PCR thresholds or blinding. The reference standard was low risk in most studies, although three studies were rated high due to incomplete alignment with accepted PJP definitions. Applicability concerns were mainly related to patient selection (5/12 studies) and the reference standard (3/12 studies), while the index test was consistently applicable (Tables S7 and S8 in the Electronic Supplementary Materials).

Finally, Deeks’ funnel plot asymmetry test indicated no significant evidence of publication bias (*p* = 0.626). The bias estimate (5.23, SE = 10.41) was not statistically significant, and visual inspection of the funnel plot revealed no major asymmetry, suggesting that small-study effects or selective reporting were unlikely to have substantially influenced the pooled diagnostic estimates.

## 4. Discussion

This systematic review demonstrates the significant role that the use of PCR detection in oropharyngeal wash samples represents as a non-invasive tool for the diagnosis of PJP in immunocompromised patients. Our results are similar to recent meta-analyses investigating various non-invasive tools [23,24]. The pooled sensitivity of 68.3% obtained proposes OW as a valuable tool, particularly when induced sputum or BAL are unavailable or not advisable [23,25]; this may be of particular interest in low-resource settings, where a large proportion of the global HIV population resides. Moreover, the pooled specificity of 91.8% clearly allows the exclusion of PJP when a negative is obtained [24], which in turn avoids further invasive testing and inadequate therapy with trimethoprim/sulfamethoxazole (and systemic steroids in the case of HIV patients). The latter two reduce antimicrobial pressure and therapy related toxicities for these fragile patients. On the other hand, a positive result supports prompt treatment initiation and may reduce mortality—clear benefits for patient care.

In the last few years, PJP diagnosis has evolved, and PCR methods in induced sputum, as well as in BAL samples may now be used, according to the most current guidelines for both HIV (CDC/NIH guidelines [8]) and non-HIV populations (EORT guidelines [9]). As demonstrated by our findings, OW PCR diagnosis of PJP also presents as a safe and accurate alternative. Among the twelve studies included, only eight articles [13,16,17,18,19,20,21,22] have explicitly stated ethical board approval and details on patient informed consent; the remaining studies correspond to older publications.

Based on this review, the authors suggest a number of recommendations as guidelines for the clinical management: (1) Screening—OW PCR may serve as an initial test in moderate-to-high risk patients when other sampling methods are not feasible; (2) Positive result—A positive OW PCR, particularly when using qPCR to exclude colonization, may justify early treatment while waiting for definitive diagnosis; (3) Quantitative testing—Use of quantitative PCR methods is recommended to improve diagnostic accuracy; (4) Negative result—In stable and non-high-risk patients, a negative result may rule out infection; however, if clinical suspicion remains high, further diagnostic work-up is warranted. Additionally, the collection technique depicted in Figure 2 may represent the most pragmatic and secure method. All of these recommendations must be taken into consideration cautiously, as further studies to confirm the diagnostic accuracy of this sampling method are required.

Strengths of this systematic review include a focused research question addressing a clinically relevant and present topic, PRISMA guideline adherence with a prospective search strategy, and prospective protocol registration in the PROSPERO database, thus providing scientific transparency, minimizing the risk of selective reporting and enhancing reproducibility. Moreover, the application of strict inclusion criteria ensured comparable patient populations and outcomes, strengthening the validity of pooled estimates. Furthermore, quality appraisal through the QUADAS-2 tool plainly documented risk of bias and applicability concerns, where most studies were found to be low risk in key domains, adding credibility to the synthesized results. The inclusion of safety analysis and identification of the influence of sample collection factors (e.g., gargling duration, cough induction, timing relative to treatment initiation) on diagnostic yield, adds practical, procedure-specific insights. The meta-analysis applied a bivariate random-effects model (Reitsma model) to jointly estimate pooled sensitivity and specificity, appropriately accounting for between-study heterogeneity. The inclusion of summary ROC curves, heterogeneity statistics, and publication bias testing (Deeks’ test) reflects a high level of statistical thoroughness. Lastly, clinical recommendations applicable in low-resource settings and with the potential to guide early treatment initiation, were provided.

On the other hand, regarding limitations, the number of included articles was small, hampering the ability to characterize diagnostic performance. Despite its suggested use since the early 1990s, OW PCR remains relatively non-utilized. Furthermore, considerable methodological heterogeneity was observed across studies, particularly in PCR platforms, molecular targets and positivity thresholds—most likely attributable to evolving technological advancements. Nonetheless, these can obviously affect the diagnostic accuracy and reproducibility. Moreover, the importance of differentiating infection versus colonization in these immunosuppressed patients cannot be undermined [24,26]—thus, an important limitation of this systematic review relates to the majority of the included studies employing qualitative instead of quantitative PCR methods, which affects the diagnostic accuracy of OW PCR diagnosis of PJP. This finding may partly be explained by the inclusion of more pivotal initial studies, carried out when qPCR was yet unavailable. Moreover, in the EORTC guidelines [9] the probable diagnosis definition only includes quantitative PCR, despite the absence of a consensual threshold to distinguish between infection and colonization. Further research employing standardized qPCR with validated thresholds will be essential to determine precise diagnostic accuracy estimates for OW PCR in this setting.

Additionally, despite broadening the inclusion criteria for all immunocompromised patients, the great majority of the papers focused on HIV patients. As stated before, the epidemiology of PJP is transforming by moving away from untreated and severe cases of HIV and AIDS patients, to targeting other immunocompromised groups either by disease (haematological malignancies and others) or by pharmacological intervention (systemic corticosteroids, immunomodulators, etc.) [27,28,29]. Advancements in treatment and prophylaxis in HIV patients, along with expanded utilization of innovative immunosuppressive therapies for a growing number of systemic diseases, are mostly responsible for this epidemiological shift. Despite the current borrowing of PJP management strategies from HIV to non-HIV patients, studies have demonstrated that PJP presentation may be quite distinct, namely in mortality. Several knowledge gaps in non-HIV patients remain, including prophylaxis duration and regimen, differentiation of colonization versus infection and the removal of corticosteroid as adjunctive treatment [30,31]. Ultimately, this growing at-risk PJP population demand for a targeted approach. Therefore, additional research on the performance of this diagnostic tool in non-HIV populations is essential to confirm the findings of this systematic review.

While our findings highlight the method’s clinical utility, they also reveal current limitations—including heterogeneity in PCR methodologies, variable reporting of pre-analytical conditions, and an overrepresentation of HIV-positive cohorts. Overall, despite acceptable methodological quality, variability in patient selection and reference standard criteria should be considered when interpreting pooled diagnostic performance. Hence, expanding research into non-HIV immunosuppressed populations and standardizing collection and analysis protocols will be critical for validating and optimizing this technique.

Ultimately, in an era where rapid, patient-friendly diagnostics are increasingly essential, OW-based PCR has the potential to improve timely PJP detection, guide early therapeutic decisions, and ultimately reduce morbidity and mortality in vulnerable patient groups.

## 5. Conclusions

This systematic review demonstrates that PCR detection of *Pneumocystis jirovecii* in oropharyngeal wash samples offers a promising, safe, and non-invasive approach for diagnosing PJP in immunocompromised patients. With a moderate pooled sensitivity of 68.3% and high specificity of 91.8%, OW-based PCR provides clinically meaningful diagnostic accuracy, particularly valuable when bronchoalveolar lavage or induced sputum is unavailable, unsafe, or impractical. Its excellent safety profile, procedural simplicity, and potential applicability in both high-resource and low-resource settings further support its role as an initial screening tool or adjunctive test in PJP diagnostic pathways.

## Figures and Tables

**Table 1:** Summarized table of the twelve studies included in the systematic review. A detailed version of this table is available in Table S6 in the Electronic Supplementary Materials. FN—False negative; FP—False positive; HIV—Human immunodeficiency virus; N—No; OW—Oropharyngeal wash; P—Prospective; PCR—Polymerase chain reaction; PJP—*Pneumocystis jirovecii* pneumonia; R—Retrospective; TN—True negative; TP—True positive; Y—Yes.

Authors	Study Design	Total Patients/PJP Cases	Immunosuppression (Except HIV)	HIV	OW Sample	Qualitative PCR	Quantitative PCR	TP	FP	TN	FN
Lundgren et al. [11]	P	49/25	N	Y	Y	Y	N	18	1	23	7
Tamburrini et al. [12]	P	18/18	N	Y	Y	Y	N	15	0	18	8
Atzori et al. [13]	P	27/14	N	Y	Y	Y	N	11	0	13	3
Helweg-Larsen J. et al. [14]	P	76/28	N	Y	Y	Y	N	25	2	31	3
O. Matos et al. [15]	P	104/52	N	Y	Y	Y	N	26	0	32	26
Goterris L. et al. [16]	R	36/15	Y	Y	Y	Y	Y	5	0	22	0
Huang L. et al. [17]	P	34/21	N	Y	Y	N	Y	13	0	15	8
Larsen et al. [18]	P	108/65	N	Y	Y	N	Y, semi-quantitative	20	14	77	3
Nyamande et al. [19]	P	35/16	N	Y	Y	Y	N	14	3	10	21
Oliveira A. et al. [20]	P	58/37	N	Y	Y	Y	N	13	2	19	24
Juliano J. et al. [21]	R	63/45	N	Y	Y	Y	Y	45	0	16	16
Halsema C. et al. [22]	P	45/27	Y	Y	Y	Y	N	8	0	20	2

## Data Availability

Data available upon reasonable request to the corresponding author.

## Supplementary Materials

The following supporting information can be downloaded at: https://www.mdpi.com/article/10.3390/jcm14186572/s1. The Electronic Supplementary Materials document contains: the full search strategy (Tables S1–S5), a detailed summary of the included studies in this systematic review (Table S6) as well as the information regarding the QUADAS-2 risk of bias assessment (Tables S7 and S8).

## References

[1] Jaramillo Cartagena A., Asowata O.E., Ng D., Babady N.E. (2025). An Overview of the Laboratory Diagnosis of *Pneumocystis jirovecii* Pneumonia. J. Clin. Microbiol..

[2] Morris A., Norris K.A. (2012). Colonization by *Pneumocystis jirovecii* and Its Role in Disease. Clin. Microbiol. Rev..

[3] McDonald E.G., Afshar A., Assiri B., Boyles T., Hsu J.M., Khuong N., Prosty C., So M., Sohani Z.N., Butler-Laporte G. (2024). *Pneumocystis jirovecii* Pneumonia in People Living with HIV: A Review. Clin. Microbiol. Rev..

[4] Thomas C.F., Limper A.H. (2004). *Pneumocystis* Pneumonia. N. Engl. J. Med..

[5] Lécuyer R., Issa N., Camou F., Lavergne R., Gabriel F., Morio F., Canet E., Raffi F., Boutoille D., Cady A. (2024). Characteristics and Prognosis Factors of *Pneumocystis jirovecii* Pneumonia According to Underlying Disease. Chest.

[6] Limper A.H., Offord K.P., Smith T.F., Martin W.J. (1989). *Pneumocystis carinii* Pneumonia: Differences in Lung Parasite Number and Inflammation in Patients with and without AIDS. Am. Rev. Respir. Dis..

[7] Lagrou K., Chen S., Masur H., Viscoli C., Decker C.F., Pagano L., Groll A.H. (2021). *Pneumocystis jirovecii* Disease: Basis for the Revised EORTC/MSGERC Invasive Fungal Disease Definitions in Individuals Without Human Immunodeficiency Virus. Clin. Infect. Dis..

[8] National Institutes of Health, Centers for Disease Control and Prevention, HIV Medicine Association, Infectious Diseases Society of America Guidelines for the Prevention and Treatment of Opportunistic Infections in Adults and Adolescents with HIV. https://clinicalinfo.hiv.gov/en/guidelines/hiv-clinical-guidelines-adult-and-adolescent-opportunistic-infections/whats-new.

[9] Donnelly J.P., Chen S.C., Kauffman C.A., Steinbach W.J., Baddley J.W., Verweij P.E., Clancy C.J., Wingard J.R., Lockhart S.R., Groll A.H. (2020). Revision and Update of the Consensus Definitions of Invasive Fungal Disease from the European Organization for Research and Treatment of Cancer and the Mycoses Study Group Education and Research Consortium. Clin. Infect. Dis..

[10] Del Corpo O., Butler-Laporte G., Sheppard D.C., Cheng M.P., McDonald E.G., Lee T.C. (2020). Diagnostic Accuracy of Serum (1-3)-β-D-Glucan for *Pneumocystis jirovecii* Pneumonia: A Systematic Review and Meta-Analysis. Clin. Microbiol. Infect..

[11] Lundgren B., Benfield T., Lundgren J.D. (1996). Evaluation of PCR Technique for Diagnosing *Pneumocystis carinii* Pneumonia in HIV Positive Patients Using Oropharyngeal Washings. J. Eukaryot. Microbiol..

[12] Tamburrini E., Ortona E., Visconti E., Margutti P., Mencharini P., Zolefo M., Marinaci S., Siracusano A. (1997). Detection of *Pneumocystis carinii* in Oropharyngeal Washings by PCR-SHELA and Nested PCR. J. Eukaryot. Microbiol..

[13] Atzori C., Agostoni F., Angeli E., Mainini A., Orlando G., Cargnel A. (1998). Combined Use of Blood and Oropharyngeal Samples for Noninvasive Diagnosis of *Pneumocystis carinii* Pneumonia Using the Polymerase Chain Reaction. Eur. J. Clin. Microbiol. Infect. Dis..

[14] Helweg-Larsen J., Skov Jensen J., Benfield T., Gerner Svendsen U., Lundgren J.D., Lundgren B. (1998). Diagnostic Use of PCR for Detection of *Pneumocystis carinii* in Oral Wash Samples. J. Clin. Microbiol..

[15] Matos O., Costa M., Lundgren B., Caldeira L., Aguiar P., Antunes F. (2001). Effect of Oral Washes on the Diagnosis of *Pneumocystis carinii* Pneumonia with a Low Parasite Burden and on Detection of Organisms in Subclinical Infections. Eur. J. Clin. Microbiol. Infect. Dis..

[16] Goterris L., Mancebo Fernández M.A., Aguilar-Company J., Falcó V., Ruiz-Camps I., Martín-Gómez M.T. (2019). Molecular Diagnosis of *Pneumocystis jirovecii* Pneumonia by Use of Oral Wash Samples in Immunocompromised Patients: Usefulness and Importance of the DNA Target. J. Clin. Microbiol..

[17] Huang L., Crothers K., DeOLIVEIRA A., Eiser S., Zucchi P., Beard C.B., Unnasch T.R. (2003). Application of an mRNA-Based Molecular Viability Assay to Oropharyngeal Washes for the Diagnosis of *Pneurnocystis* Pneumonia in HIV-Infected Patients. A Pilot Study. J. Eukaryot. Microbiol..

[18] Larsen H.H., Huang L., Kovacs J.A., Crothers K., Silcott V.A., Morris A., Turner J.R., Beard C.B., Masur H., Fischer S.H. (2004). A Prospective, Blinded Study of Quantitative Touch-Down Polymerase Chain Reaction Using Oral-Wash Samples for Diagnosis of *Pneumocystis* Pneumonia in HIV-Infected Patients. J. Infect. Dis..

[19] Nyamande K., Lalloo U.G., York D., Naidoo M., Irusen E.M., Chetty R. (2005). Low Sensitivity of a Nested Polymerase Chain Reaction in Oropharyngeal Washings for the Diagnosis of *Pneumocystis* Pneumonia in HIV-Infected Patients. Chest.

[20] De Oliveira A., Unnasch T.R., Crothers K., Eiser S., Zucchi P., Moir J., Beard C.B., Lawrence G.G., Huang L. (2007). Performance of a Molecular Viability Assay for the Diagnosis of *Pneumocystis* Pneumonia in HIV-Infected Patients. Diagn. Microbiol. Infect. Dis..

[21] Juliano J.J., Barnett E., Parobek C.M., Taylor S.M., Meshnick S.R., Stone S., Chang E., Fong S., Huang L. (2015). Use of Oropharyngeal Washes to Diagnose and Genotype *Pneumocystis jirovecii*. Open Forum Infect. Dis..

[22] Van Halsema C., Johnson L., Baxter J., Douthwaite S., Clowes Y., Guiver M., Ustianowski A. (2016). Short Communication: Diagnosis of *Pneumocystis jirovecii* Pneumonia by Detection of DNA in Blood and Oropharyngeal Wash, Compared with Sputum. AIDS Res. Hum. Retrovir..

[23] Senécal J., Smyth E., Del Corpo O., Hsu J.M., Amar-Zifkin A., Bergeron A., Cheng M.P., Butler-Laporte G., McDonald E.G., Lee T.C. (2022). Non-Invasive Diagnosis of *Pneumocystis jirovecii* Pneumonia: A Systematic Review and Meta-Analysis. Clin. Microbiol. Infect..

[24] Brown L., Rautemaa-Richardson R., Mengoli C., Alanio A., Barnes R.A., Bretagne S., Chen S.C.A., Cordonnier C., Donnelly J.P., Heinz W.J. (2024). Polymerase Chain Reaction on Respiratory Tract Specimens of Immunocompromised Patients to Diagnose *Pneumocystis* Pneumonia: A Systematic Review and Meta-Analysis. Clin. Infect. Dis..

[25] Lu Y., Ling G., Qiang C., Ming Q., Wu C., Wang K., Ying Z. (2011). PCR Diagnosis of *Pneumocystis* Pneumonia: A Bivariate Meta-Analysis. J. Clin. Microbiol..

[26] Zhang L., Zheng C., Sun Y., Chen X., Wang Y., Xiang H., Liang Y., Wei F., Zhang Y. (2025). Diagnostic Tests Performance in Detecting *Pneumocystis jirovecii*: A Systematic Review and Meta-Analysis. Eur. J. Clin. Microbiol. Infect. Dis..

[27] Kolbrink B., Scheikholeslami-Sabzewari J., Borzikowsky C., Von Samson-Himmelstjerna F.A., Ullmann A.J., Kunzendorf U., Schulte K. (2022). Evolving Epidemiology of *Pneumocystis* Pneumonia: Findings from a Longitudinal Population-Based Study and a Retrospective Multi-Center Study in Germany. Lancet Reg. Health—Eur..

[28] Quigley N., d’Amours L., Gervais P., Dion G. (2024). Epidemiology, Risk Factors, and Prophylaxis Use for *Pneumocystis jirovecii* Pneumonia in the Non-HIV Population: A Retrospective Study in Québec, Canada. Open Forum Infect. Dis..

[29] Kao T.-W., Ruan S.-Y., Huang Y.-T., Liu W.-D., Liu C.-J., Chen Y.-Y., Hsueh P.-R., Yu C.-J., Chien J.-Y. (2025). Evolving Risk Factors and Predisposing Conditions of *Pneumocystis* Pneumonia in Non-HIV Patients: A Seven-Year Multicenter Study. J. Infect..

[30] Kamel T., Janssen-Langenstein R., Quelven Q., Chelly J., Valette X., Le M.-P., Bourenne J., Garot D., Fillatre P., Labruyere M. (2024). *Pneumocystis* Pneumonia in Intensive Care: Clinical Spectrum, Prophylaxis Patterns, Antibiotic Treatment Delay Impact, and Role of Corticosteroids. A French Multicentre Prospective Cohort Study. Intensive Care Med..

[31] Lemiale V., Resche-Rigon M., Zerbib Y., Mokart D., De Prost N., Wallet F., Perez P., Kouatchet A., Argaud L., Decavèle M. (2025). Adjunctive Corticosteroids in Non-AIDS Patients with Severe *Pneumocystis jirovecii* Pneumonia (PIC): A Multicentre, Double-Blind, Randomised Controlled Trial. Lancet Respir. Med..

