# Supplementary material for: Pneumocystis jirovecii Pneumonia Diagnosis with Oropharyngeal Wash PCR in Immunocompromised Patients—A Systematic Review
Source: J Clin Med. 2025 Sep 18;14(18):6572. doi: 10.3390/jcm14186572 (PMC12470813; doi:10.3390/jcm14186572)
Supplement: Supplementary file 1 [file jcm-14-06572-s001.zip › jcm-3846078-supplementary.pdf]

Electronic Supplementary Materials

Search Strategies – Tables S1 to S5

Table S1. Pubmed Search Strategy. OPW – oropharyngeal wash; PCP – *Pneumocystis carinii* pneumonia; PCR – Polymerase chain reaction; qRT-PCR – Quantitative real-time PCR; RT-PCR – Real-time PCR.

#	Query	Results
1	"Pneumocystis jirovecii" OR "P. jirovecii Pneumonia" OR "Pneumocystis carinii" OR "P. carinii Pneumonia" OR "Pneumocystis pneumonia" OR "PcP"	28 592
2	mouthwash* OR "oral wash*" OR "oral rinse*" OR "mouth rinse*" OR "oral lavage*" OR "oral fluid*" OR "saliva sample*" OR "oral sample*" OR "oropharyngeal wash*" OR OPW	25 317
3	PCR OR "polymerase chain reaction" OR "molecular diagnosis*" OR "nucleic acid amplification" OR "RT-PCR" OR "qRT-PCR" OR "quantitative real-time PCR"	1 046 718
4	#1 AND #2 AND #3	48

Table S2. Scopus Search Strategy. OPW – oropharyngeal wash; PCP – *Pneumocystis carinii* pneumonia; PCR – Polymerase chain reaction; qRT-PCR – Quantitative real-time PCR; RT-PCR – Real-time PCR.

#	Query	Results
1	"Pneumocystis jirovecii" OR "P. jirovecii Pneumonia" OR "Pneumocystis carinii" OR "P. carinii Pneumonia" OR "Pneumocystis pneumonia" OR "PcP"	45 338
2	mouthwash* OR "oral wash*" OR "oral rinse*" OR "mouth rinse*" OR "oral lavage*" OR "oral fluid*" OR "saliva sample*" OR "oral sample*" OR "oropharyngeal wash*" OR OPW	32 120
3	PCR OR "polymerase chain reaction" OR "molecular diagnosis*" OR "nucleic acid amplification" OR "RT-PCR" OR "qRT-PCR" OR "quantitative real-time PCR"	1 671 568
4	#1 AND #2 AND #3	57

Table S3. Web of Science Search Strategy. OPW – oropharyngeal wash; PCP – *Pneumocystis carinii* pneumonia; PCR – Polymerase chain reaction; qRT-PCR – Quantitative real-time PCR; RT-PCR – Real-time PCR.

#	Query	Results
1	"Pneumocystis jirovecii" OR "P. jirovecii Pneumonia" OR "Pneumocystis carinii" OR "P. carinii Pneumonia" OR "Pneumocystis pneumonia" OR "PcP"	32 789
2	mouthwash* OR "oral wash*" OR "oral rinse*" OR "mouth rinse*" OR "oral lavage*" OR "oral fluid*" OR "saliva sample*" OR "oral sample*" OR "oropharyngeal wash*" OR OPW	23 047
3	PCR OR "polymerase chain reaction" OR "molecular diagnosis*" OR "nucleic acid amplification" OR "RT-PCR" OR "qRT-PCR" OR "quantitative real-time PCR"	943 820
4	#1 AND #2 AND #3	53

Table S4. Cochrane Library Search Strategy. OPW – oropharyngeal wash; PCP – *Pneumocystis carinii* pneumonia; PCR – Polymerase chain reaction; qRT-PCR – Quantitative real-time PCR; RT-PCR – Real-time PCR.

#	Query	Results (trials)
1	"Pneumocystis jirovecii" OR "P. jirovecii Pneumonia" OR "Pneumocystis carinii" OR "P. carinii Pneumonia" OR "Pneumocystis pneumonia" OR "PcP"	1785
2	mouthwash OR "oral wash" OR "oral rinse" OR "mouth rinse" OR "oral lavage" OR "oral fluid" OR "saliva sample" OR "oral sample" OR "oropharyngeal wash" OR OPW	8057
3	PCR OR "polymerase chain reaction" OR "molecular diagnosis*" OR "nucleic acid amplification" OR "RT-PCR" OR "qRT-PCR" OR "quantitative real-time PCR"	22 805
4	#1 AND #2 AND #3	14

Table S5. Trials Registers Search Strategy. OPW – oropharyngeal wash; PCP – *Pneumocystis carinii* pneumonia; PCR – Polymerase chain reaction; qRT-PCR – Quantitative real-time PCR; RT-PCR – Real-time PCR.

	Query	Results
EU Clinical Trials Register	("Pneumocystis jirovecii" OR "P. jirovecii Pneumonia" OR "Pneumocystis carinii" OR "P. carinii Pneumonia" OR "Pneumocystis pneumonia" OR "PcP") AND ("mouthwash" OR "oral wash" OR "oral rinse*" OR "mouth rinse" OR "oral lavage" OR "oral fluid*" OR "saliva sample*" OR "oral sample*" OR "oropharyngeal wash* OR OPW) AND (PCR OR "polymerase chain reaction" OR "molecular diagnosis*" OR "nucleic acid amplification" OR "RT-PCR" OR "qRT-PCR" OR "quantitative real-time PCR")	0
ICTRP	("Pneumocystis jirovecii" OR "P. jirovecii Pneumonia" OR "Pneumocystis carinii" OR "P. carinii Pneumonia" OR "Pneumocystis pneumonia" OR "PcP") AND ("mouthwash" OR "oral wash" OR "oral rinse*" OR "mouth rinse" OR "oral lavage" OR "oral fluid*" OR "saliva sample*" OR "oral sample*" OR "oropharyngeal wash* OR OPW) AND (PCR OR "polymerase chain reaction" OR "molecular diagnosis*" OR "nucleic acid amplification" OR "RT-PCR" OR "qRT-PCR" OR "quantitative real-time PCR")	0
ISRCTN	("Pneumocystis jirovecii" OR "P. jirovecii Pneumonia" OR "Pneumocystis carinii" OR "P. carinii Pneumonia" OR "Pneumocystis pneumonia" OR "PcP") AND ("mouthwash" OR "oral wash" OR "oral rinse*" OR "mouth rinse" OR "oral lavage" OR "oral fluid*" OR "saliva sample*" OR "oral sample*" OR "oropharyngeal wash* OR OPW) AND (PCR OR "polymerase chain reaction" OR "molecular diagnosis*" OR "nucleic acid amplification" OR "RT-PCR" OR "qRT-PCR" OR "quantitative real-time PCR")	0
EuropePMC	("Pneumocystis jirovecii" OR "P. jirovecii Pneumonia" OR "Pneumocystis carinii" OR "P. carinii Pneumonia" OR "Pneumocystis pneumonia" OR "PcP") AND ("mouthwash" OR "oral wash" OR "oral rinse*" OR "mouth rinse" OR "oral lavage" OR "oral fluid*" OR "saliva sample*" OR "oral sample*" OR "oropharyngeal wash* OR OPW) AND (PCR OR "polymerase chain reaction" OR "molecular diagnosis*" OR "nucleic acid amplification" OR "RT-PCR" OR "qRT-PCR" OR "quantitative real-time PCR")	0
Clinicaltrials.gov	("Pneumocystis jirovecii" OR "P. jirovecii Pneumonia" OR "Pneumocystis carinii" OR "P. carinii Pneumonia" OR "Pneumocystis pneumonia" OR "PcP") AND (mouth OR oral OR saliva OR oropharyngeal) AND (PCR OR "polymerase chain reaction" OR "molecular diagnosis*" OR "nucleic acid amplification" OR "RT-PCR" OR "qRT-PCR" OR "quantitative real-time PCR")	17
Preprints.org	("Pneumocystis jirovecii" OR "P. jirovecii Pneumonia" OR "Pneumocystis carinii" OR "P. carinii Pneumonia" OR "Pneumocystis pneumonia" OR "PcP") AND ("mouthwash" OR "oral wash" OR "oral rinse*" OR "mouth rinse" OR "oral lavage" OR "oral fluid*" OR "saliva sample*" OR "oral sample*" OR "oropharyngeal wash* OR OPW) AND (PCR OR "polymerase chain reaction" OR "molecular diagnosis*" OR "nucleic acid amplification" OR "RT-PCR" OR "qRT-PCR" OR "quantitative real-time PCR")	0
MedRxiv	(Pneumocystis jirovecii" OR "Pneumocystis carinii") AND (oral OR saliva) AND PCR" (match all words)	39
SSRN	("Pneumocystis jirovecii" OR "Pneumocystis carinii" OR "Pneumocystis pneumonia" OR "PcP") AND (mouth OR oral OR saliva OR oropharyngeal) AND (PCR OR "polymerase chain reaction")	0
OSF	("Pneumocystis jirovecii" OR "Pneumocystis carinii" OR "Pneumocystis pneumonia" OR "PcP") AND (mouth OR oral OR saliva OR oropharyngeal) AND (PCR OR "polymerase chain reaction")	0

Table S6. Description of included studies. ART – Antiretroviral therapy, BAL – Bronchoalveolar lavage, FN – False negative, FP – False positive, HIV – Human immunodeficiency virus, IS – Induced sputum, mL – milliliters, N – No, PCR – Polymerase chain reaction, PCR-SHELA – Polymerase chain reaction-solution hybridization enzyme-linked immunoassay, PJP – *Pneumocystis jirovecii* pneumonia, TN – True negative, TP – True positive, Y –Yes.

**Article**	**Evaluation of PCR technique for diagnosing Pneumocystis carinii Pneumonia in HIV positive patients using oropharyngeal washings [11]**	**Detection of Pneumocystis carinii in oropharyngeal washings by PCR-SHELA and nested PCR [12]**	**Combined use of blood and oropharyngeal samples for noninvasive diagnosis of Pneumocystis carinii pneumonia using the polymerase chain reaction [13]**	**Diagnostic use of PCR for detection of Pneumocystis carinii in oral wash samples [14]**	**Effect of oral washes on the diagnosis of Pneumocystis carinii pneumonia with a low parasite burden and on detection of organisms in subclinical infections [15]**	**Molecular diagnosis of Pneumocystis jirovecii Pneumonia (PJP) using oral wash (OW) samples: usefulness and importance of the PCR target [16]**	**Application of an mRNA-based molecular viability assay to oropharyngeal washes for the diagnosis of Pneumocystis pneumonia in HIV-infected patients.: A pilot study [17]**	**A prospective, blinded study of quantitative touch-down polymerase chain reaction using oral-wash samples for diagnosis of Pneumocystis pneumonia in HIV-infected patients [18]**	**Low sensitivity of a nested polymerase chain reaction in oropharyngeal washings for the diagnosis of Pneumocystis pneumonia in HIV-infected patients [19]**	**Performance of a molecular viability assay for the diagnosis of Pneumocystis pneumonia in HIV-infected patients [20]**	**Use of Oropharyngeal Washes to Diagnose and Genotype Pneumocystis jirovecii [21]**	**Diagnosis of Pneumocystis jirovecii Pneumonia by Detection of DNA in Blood and Oropharyngeal Wash, Compared with Sputum [22]**
**Authors**	Lundgren et al.	Tamburrini et al.	Atzori et al.	Helweg-Larsen J. et al.	O. Matos et al.	Goterris L. et al.	Huang L. et al.	Larsen et al.	Nyamande et al.	Oliveira A. et al.	Juliano J. et al.	Halsema C. et al.
**Year**	1996	1997	1998	1998	2001	2019	2003	2004	2005	2006	2015	2016
**Study**	Prospective	Prospective	Prospective	Prospective	Prospective	Retrospective	Prospective	Prospective	Prospective	Prospective	Retrospective	Prospective
**Total number of patients included/Number of PJP confirmed cases**	49/25	18/18	27/14	76/28	104/52	36/15	34/21	108/65	35/16	58/37	63/45	45/27
**Immunosuppression (apart from HIV)**	N	N	N	N	N	Y	N	N	N	N	N	Y
**HIV (Y/N)**	Y	Y	Y	Y	Y	Y	Y	Y	Y	Y	Y	Y

**Mention of ART therapy for HIV (Y/N)**	N	N	N	N	N	N	N	N	N	N	N	Y
**Mention of ART compliance (Y/N)**	N	N	N	N	N	N	N	N	Y	N	N	N
**Other immunosuppressive therapy (Y/N)**	N	N	N	N	N	Y	N	N	N	N	N	N
**If yes, which therapy**	-	-	-	-	-	Systemic corticosteroids	-	-	-	-	-	-
**Clinical and/or imaging criteria for PJP (Y/N)**	N	Y	Y	N	Y	Y	N	N	N	Y	Y	Y
**Microbiological criteria for PJP (Y/N)**	Y	Y	Y	Y	Y	Y	Y	Y	Y	Y	Y	Y
**Oropharyngeal wash sample (Y/N)**	Y	Y	Y	Y	Y	Y	Y	Y	Y	Y	Y	Y
**Sample volume (mL)**	10 mL	10 mL	10 mL	10 mL	10 mL	10 mL	10 mL	10 mL	10-20 mL	10 mL	10 mL	10 mL
**Number of gargles and gargling time (N and seconds)**	-/ -	-/-	-/120 seconds	-/60 seconds	-/60 seconds	Vigorous gargling/30 seconds	-/Up to 60 seconds	-/60 seconds	-/5 seconds	-/60 seconds	-/60 seconds	-/Between 10-30 seconds
**Sample storage**	-	-	-20°C aliquots	-	-	-	-	-	4°C before sample processing	-70°C before shipping	-80°C before shipping	-
**Sample transportation**	-	-	-	-	-	-	Transport on dry ice	-	-	-	-	-
**Safety measures**	-	-	No complications	No complications	No complications	-	-	-	-	-	-	-
**Other respiratory sample excluding BAL (Y/N)**	N	Y	N	N	Y	N	Y	Y	N	Y	Y	Y

**Which respiratory sample**	-	IS	-	-	IS	-	IS	IS	-	IS	IS	IS
**Qualitative PCR (Y/N)**	Y	Y	Y	Y	Y	Y	N	N	Y	Y	Y	Y
**Quantitative PCR (Y/N)**	N	N	N	N	N	Y	Y	Y, semi-quantitative	N	N	Y	N
**Exclusion of colonization (Y/N)**	N	N	N	N	N	Y	N	N	N	N	Y	Y
**Serum Beta-D-glucan (Y/N)**	N	N	N	N	N	N	N	N	N	N	N	N
**Respiratory sample Beta-D-glucan (Y/N)**	N	N	N	N	N	N	N	N	N	N	N	N
**TP**	18	15	11	25	26	5	13	20	14	13	45	8
**FP**	1	0	0	2	0	0	0	14	3	2	0	0
**TN**	23	18	13	31	32	22	15	77	10	19	16	20
**FN**	7	8	3	3	26	0	8	3	21	24	16	2

Table S7. Customized QUADAS-2 Checklist for Review. EORTC – European Organisation For Research and Treatment of Cancer; N/A – not available; PCR – Polymerase chain reaction; PJP – *Pneumocystis jirovecii* pneumonia; QUADAS-2 – Quality Assessment of Diagnostic Accuracy Studies-2.

Domain	Signaling Questions	Risk of Bias Judgement	Applicability Concerns
**Patient Selection**	- Were patients enrolled consecutively or randomly? - Were any inappropriate exclusions made?	Low / High / Unclear	Is the study population representative of immuno-compromised adults with suspected PJP?
**Index Test**	- Was PCR performed and interpreted without knowledge of the reference standard? - Was the PCR method standardized and consistent?	Low / High / Unclear	Is the index test (PCR on mouthwash/oropharyngeal samples) conducted as per your review focus?
**Reference Standard**	- Is EORTC criteria used as the reference standard? - Was it applied independently of the PCR results?	Low / High / Unclear	Does the reference standard accurately define PJP diagnosis relevant to your review question?
**Flow and Timing**	- Was the time interval between PCR and reference standard appropriate? - Were all patients included in the analysis?	Low / High / Unclear	N/A (usually not applicable for applicability concerns)

Table S8. Risk of bias assessment using QUADAS-2 Checklist. ID – Identification; QUADAS-2 – Quality Assessment of Diagnostic Accuracy Studies-2.

Study ID	Patient Selection Risk	Index Test Risk	Reference Standard Risk	Flow & Timing Risk	Patient Selection Applicability	Index Test Applicability	Reference Standard Applicability
**Betiina Lundgren, et al.**	Low	Low	High	Low	High	Low	Unclear
**Enrica Tamburrini, et al.**	Low	High	Low	Low	High	Low	Low
**C. Atzori, et al.**	Low	Low	Low	Low	Low	Low	Low
**Jannik Helweg-Larsen, et al.**	Low	Low	Low	Low	High	Low	Unclear
**O. Matos, et al.**	Low	Low	Low	Low	Low	Low	Low
**Lidia Goterris, et al.**	High	Low	Low	Low	High	Low	High
**Laurence Huang, et al.**	High	Low	High	Low	Unclear	Low	High
**Hans Henrik Larsen, et al.**	Low	Low	Low	Low	Low	Low	Low
**Kennedy Nyamande, et al.**	High	Low	Low	Low	Unclear	Low	Low

**Ana de Oliveira, et al.**	Low	Low	Low	Low	Low	Low	Low
**Jonathan J. Juliano, et al.**	Low	Low	Low	Low	Low	Low	Low
**Clare van Halsema, et al.**	High	Low	High	Low	High	Low	High
